# Interplay between low threshold voltage-gated K^+^ channels and synaptic inhibition in neurons of the chicken nucleus laminaris along its frequency axis

**DOI:** 10.3389/fncir.2014.00051

**Published:** 2014-05-21

**Authors:** William R. Hamlet, Yu-Wei Liu, Zheng-Quan Tang, Yong Lu

**Affiliations:** ^1^Department of Anatomy and Neurobiology, College of Medicine, Northeast Ohio Medical UniversityRootstown, OH, USA; ^2^School of Biomedical Sciences, Kent State UniversityKent, OH, USA

**Keywords:** GABAergic inhibition, voltage-gated low-threshold potassium current, IPSC, IPSP, tonotopy, whole-cell patch, interaural time difference

## Abstract

Central auditory neurons that localize sound in horizontal space have specialized intrinsic and synaptic cellular mechanisms to tightly control the threshold and timing for action potential generation. However, the critical interplay between intrinsic voltage-gated conductances and extrinsic synaptic conductances in determining neuronal output are not well understood. In chicken, neurons in the nucleus laminaris (NL) encode sound location using interaural time difference (ITD) as a cue. Along the tonotopic axis of NL, there exist robust differences among low, middle, and high frequency (LF, MF, and HF, respectively) neurons in a variety of neuronal properties such as low threshold voltage-gated K^+^ (LTK) channels and depolarizing inhibition. This establishes NL as an ideal model to examine the interactions between LTK currents and synaptic inhibition across the tonotopic axis. Using whole-cell patch clamp recordings prepared from chicken embryos (E17–E18), we found that LTK currents were larger in MF and HF neurons than in LF neurons. Kinetic analysis revealed that LTK currents in MF neurons activated at lower voltages than in LF and HF neurons, whereas the inactivation of the currents was similar across the tonotopic axis. Surprisingly, blockade of LTK currents using dendrotoxin-I (DTX) tended to broaden the duration and increase the amplitude of the depolarizing inhibitory postsynaptic potentials (IPSPs) in NL neurons without dependence on coding frequency regions. Analyses of the effects of DTX on inhibitory postsynaptic currents led us to interpret this unexpected observation as a result of primarily postsynaptic effects of LTK currents on MF and HF neurons, and combined presynaptic and postsynaptic effects in LF neurons. Furthermore, DTX transferred subthreshold IPSPs to spikes. Taken together, the results suggest a critical role for LTK currents in regulating inhibitory synaptic strength in ITD-coding neurons at various frequencies.

## INTRODUCTION

Neurons rely on a variety of intrinsic and synaptic neuronal properties to ensure precise coding of temporal information from sensory inputs. An extensive body of research has demonstrated the prominent roles of synaptic inhibition in auditory neurons that encode the location of sound in azimuth space using interaural time difference (ITD) as a cue (e.g., Grothe and Sanes, 1993, 1994; Funabiki et al., 1998; Brand et al., 2002; Grothe, 2003; Zhou et al., 2005; Pecka et al., 2008; Fukui et al., 2010). These neurons encode sound location by producing maximal spiking activity when bilateral excitatory inputs from the two cochleae converge, a process termed coincidence detection (Jeffress, 1948; Konishi, 2003; MacLeod and Carr, 2012). Synaptic inhibition can improve coincidence detection via shunting the impact of depolarizing postsynaptic currents on the membrane potential and thus sharpening the time window for spike generation (Brückner and Hyson, 1998; Funabiki et al., 1998; Ingham and McAlpine, 2005; Howard and Rubel, 2010; Tang et al., 2011; Roberts et al., 2013).

In most mature neurons, synaptic inhibition mediated by ionotropic GABA_A_ and glycine receptors produces conventional hyperpolarizing inhibitory postsynaptic currents (IPSCs). In contrast, GABAergic and glycinergic IPSCs in chicken auditory brainstem neurons are depolarizing, caused by a depolarized reversal potential for Cl^-^ (between -45 and -35 mV) that appears to be maintained in mature animals (Hyson et al., 1995; Lu and Trussell, 2001; Monsivais and Rubel, 2001; Kuo et al., 2009; Tang et al., 2009). Of particular interest, sound-localizing neurons in the nucleus laminaris (NL) of the chick receive depolarizing inhibitory inputs originating primarily from ipsilateral superior olivary nucleus (SON) neurons and sparsely from local GABAergic neurons (von Bartheld et al., 1989; Code and Churchill, 1991; Lachica et al., 1994; Yang et al., 1999; Burger et al., 2005; Tang et al., 2011; Yamada et al., 2013). There exists a tonotopic distribution of GABAergic inhibition along the frequency axis of NL. Neurons coding low frequency (LF) sound receive small and fast phasic inhibition with minimal tonic inhibition, whereas neurons coding middle and high frequency (MF and HF, respectively) sound receive large and slow phasic inhibition with prominent tonic inhibition (Tang et al., 2011; Tang and Lu, 2012; Yamada et al., 2013). The depolarizing inhibition improves coincidence detection via both a shunting effect of the GABAergic conductance and the activation of a low threshold voltage-gated K^+^ (LTK) conductance (Funabiki et al., 1998; Monsivais et al., 2000; Howard et al., 2007; Tang et al., 2011).

LTK currents are prominent in auditory brainstem neurons involved in sound localization circuitry (Manis and Marx, 1991; Forsythe and Barnes-Davies, 1993; Reyes et al., 1994; Brew and Forsythe, 1995; Rathouz and Trussell, 1998; Golding et al., 1999; Brew et al., 2003; Barnes-Davies et al., 2004). In these neurons, LTK currents minimize the impact of small and slow excitatory postsynaptic currents (EPSCs) on membrane potential, regulate the threshold for action potential generation, and suppress hyperexcitability at presynaptic terminals (Dodson et al., 2002, 2003; Svirskis et al., 2002; Scott et al., 2005; Gittelman and Tempel, 2006; Mathews et al., 2010). Ion channels containing subunits from K_v1_, K_v4_, and K_v7_ subfamilies underlie the LTK currents (Coetzee et al., 1999; Johnston et al., 2010). In the tonotopically organized NL (Rubel and Parks, 1975), K_v1.1_and K_v1.2_ subunits show stronger anatomical expression in MF and HF neurons compared to LF neurons, and physiological data also suggests stronger LTK channel activity at rest in MF and HF neurons (Kuba et al., 2005). Therefore, LTK channels are poised to strongly affect synaptic integration of depolarizing synaptic inputs, particularly in MF and HF neurons. Given the unusual depolarizing nature of the inhibitory input to NL neurons and the robust presence of LTK channels in these neurons, it is important to determine how LTK currents interact with synaptic inhibition at subthreshold and suprathreshold membrane potentials, and how this interaction differs across the tonotopic axis.

## MATERIALS AND METHODS

### SLICE PREPARATION AND *IN VITRO* WHOLE-CELL RECORDINGS

Brainstem slices (250–300 μm in thickness) were prepared from chick embryos E17–E18 as described previously (Tang et al., 2009). An ice-cold artificial CSF (ACSF) used for dissecting and slicing the brain tissue contained the following (in millimolar): 250 glycerol, 3 KCl, 1.2 KH_2_PO_4_, 20 NaHCO_3_, 3 HEPES, 1.2 CaCl_2_, 5 MgCl_2_, and 10 dextrose (pH 7.4 when gassed with 95% O_2_ and 5% CO_2_). The procedures were approved by the Institutional Animal Care and Use Committee at Northeast Ohio Medical University, and are in accordance with National Institutes of Health policies on animal use. Slices were incubated at 34–36°C for approximately 1 h in normal ACSF containing the following (in millimolar): 130 NaCl, 26 NaHCO_3_, 3 KCl, 3 CaCl_2_, 1 MgCl_2_, 1.25 NaH_2_PO_4_, and 10 dextrose, pH 7.4. For recording, slices were transferred to a 0.5 ml chamber mounted on an upright Olympus BX51 microscope (Japan) with a 40× water-immersion objective. The chamber was continuously superfused with ACSF (1–2 ml/min) by gravity. Recordings were performed at 34–36°C, except K_v_ current recordings which were performed at 22–24°C (room temperature). Patch pipettes were drawn on an Electrode Puller PP-830 (Narishige) to 1–2 μm tip diameter using borosilicate glass micropipettes (inner diameter of 0.86 mm; outer diameter of 1.60 mm) (VWR Scientific). Electrode resistance was between 3 and 5 MΩ when filled with a solution containing the following (in millimolar): 105 K-gluconate, 35 KCl, 5 EGTA, 10 HEPES, 1 MgCl_2_, 4 ATP-Mg, and 0.3 GTP-Na, with pH of 7.2 (adjusted with KOH) and osmolarity between 280 and 290 mOsm/L. The Cl^-^ concentration (37 mM) in the internal solution approximated the physiological Cl^-^ concentration in NL neurons (Tang et al., 2009). Placement of recording electrodes was controlled by a micromanipulator NMN-25 (Narishige). The liquid junction potential was 10 mV, and data were corrected accordingly. Voltage- and current clamp experiments were performed with an AxoPatch 200B and an AxoClamp 2B amplifier, respectively (Molecular Devices). Voltage-clamp recordings were obtained at a holding potential of -60 mV. Data were low-pass filtered at 2–10 kHz and digitized with a Data Acquisition Interface ITC-18 (InstruTECH) at 20 kHz. Recording protocols were written and run using the acquisition and analysis software AxoGraph X (AxoGraph Scientific). In K_v_ current recordings, R_s_ compensation was done at approximately 75%. When Rs changed more than 25% during recording, the neuron was not included in data analysis. In current clamp experiments, bridge balance was used to compensate for the voltage drop across the electrode resistance.

All chemicals were purchased from Sigma–Aldrich except: (1*S*,2*S*)-2-[2-[[3-(1*H*-Benzimidazol-2-yl)propyl]methylamino]ethyl]-6-fluoro-1,2,3,4-tetrahydro-1-(1-methylethyl)-2-naphthalenyl methoxyacetoacetate dihydrochloride (Mibefrandil), 4-Ethylphenylamino-1,2-dimethyl-6-methylaminopyrimidinium chloride (ZD-7288), which were obtained from Tocris, and 6-imino-3-(4-methoxyphenyl)-1(6*H*)-pyridazine butanoic acid (SR95531), and 6,7-Dinitroquinoxaline-2,3-dione (DNQX) which were obtained from Abcam.

### SYNAPTIC STIMULATION AND RECORDINGS OF SYNAPTIC RESPONSES

Extracellular stimulation was performed using concentric bipolar electrodes with a tip core diameter of 127 μm (World Precision Instruments). The stimulating electrodes were placed using a Micromanipulator NMN-25 (Narishige) at the lateral fiber bundle, which carries both excitatory and inhibitory fibers. Blockade of AMPA receptors with DNQX (20 μM) completely blocks EPSCs, so all synaptic recordings were done in the presence of DNQX. Square electric pulses (0.2 ms duration) were delivered through a Stimulator A320RC (World Precision Instruments). Optimal stimulation parameters were selected for each cell to give postsynaptic potentials of maximal amplitude. In experiments designed to examine the effect of LTK currents on subthreshold responses, QX-314 (5 mM) was included in the pipette solution to block Na_v_ channels. A comparison of K_v_ recordings with and without QX-314 in the pipette solution showed little to no difference in LTK current size, although QX-314 can block other K^+^ conductances (Andrade, 1991; Alreja and Aghajanian, 1994).

### IDENTIFICATION OF TONOTOPIC CHARACTERISTIC FREQUENCY (CF) REGION

**FIGURE 1:**
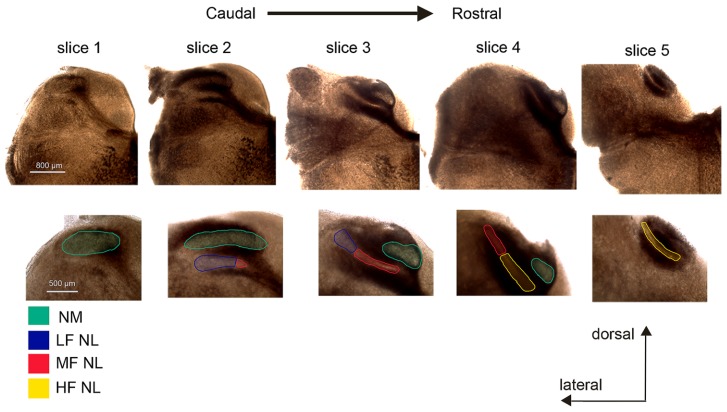
**Determination of characteristic frequency (CF) regions of nucleus laminaris (NL) in coronal brainstem slices**. Typically a total of five slices (300 μm in thickness) from each animal were collected and numbered #1 through #5, in a caudal to rostral direction. Slice #1 is characterized by the nucleus magnocellularis (NM, green) without NL. The NM is present in all but slice #5. The NL appears in slice #2 and contains mostly low frequency (LF, blue) neurons, with some middle frequency (MF, red) neurons present at the most medial end. MF neurons are most prevalent in slice #3, with LF neurons at the lateral end of the nucleus. In slice #4, HF (yellow) neurons are located in the medial portion of the NL while MF neurons are found laterally. In slice #5 only HF neurons are present. The colored boundaries between CF regions are approximate.

It is not possible to define the characteristic frequency of NL neurons in an *in vitro* slice preparation. Therefore, to categorize neurons into LF, MF, and HF regions, we used an approach modified from Kuba et al. (2005), by using the rostral–caudal and medial–lateral position as an indicator of CF. Generally five slices of brainstem tissue containing relevant nuclei were collected, and the most caudal one was slice #1 and the most rostral one slice #5. Neurons in the lateral NL of slices #2 and #3 were considered LF neurons. MF neurons were considered to be present in the medial NL of slice #2 and #3 and the lateral portion of slice #4. HF neurons were found in the medial portion of slice #4 and in slice #5 (**Figure 1**). Images were taken with a Provis AX70 (Olympus) microscope using SPOT software (Diagnostic Instruments), from freshly sliced tissue. Image contrast and colorization was adjusted using Creative Suites v2.0 (Adobe). Because boundaries between the regions are subjective, we recorded from neurons clearly present in one of the three CF regions.

### DATA ANALYSIS

The resting membrane potential was read from the amplifiers immediately after the whole-cell configuration was established. The input resistance was calculated from the voltage responses to a somatic current injection (50 pA). Current density was calculated by normalizing the raw current to each cell’s membrane capacitance, obtained during C_m_ compensation prior to recording. For K_v_ current recordings, leak subtraction was performed using a linear fitting to V_command_ -100 to -70 mV. The amount of inactivated current was calculated by subtracting the maximum current (I_max_) evoked during the V_command_ from the minimum current (I_min_) evoked during the V_command_. Current activation was measured by a single exponential function, *f*(*t*) = A*exp(-*t*/decay τ ), in which *t* stands for time and τ for time constant. Spontaneous synaptic events were detected by a template using a function for product of exponentials, *f*(*t*) = [1 - exp(-*t*/rise time)]*exp(-*t*/decay τ ). Due to differences in synaptic event size and shape within the NL, the values of these parameters for the template were determined based on the average of real events from individual cells. The detection threshold was three to four times the noise standard deviation, which allowed for a detection rate with the least number of false positives. Graphs were made in Igor (Wavemetrics). Means and SEMs are reported. Prior to hypothesis testing, normality and outliers were observed within each set of data to determine the appropriate statistical test. Outliers (>3× larger than the interquartile range) were dropped from the dataset. Wilks-Shapiro test was used to confirm whether sample distributions were approximately normally distributed. Violations of normality were present when *p* < 0.05. When significant violations of normality were present or where datasets contained *n*_i_ ≤ 7, nonparametric inferential statistical tests were used. Statistical differences were determined by Analysis of Variance (ANOVA) and Kruskal–Wallis test for parametric and nonparametric samples, respectively. When significant group differences were found, a Tukey’s *post hoc* test or Mann–Whitney *U*-test was conducted. Paired-sample *t*-tests were conducted for repeated-measures sample comparisons. Alpha-levels were corrected using the Holm–Bonferroni method.

## RESULTS

Nucleus laminaris neurons were categorized into three groups based on CF region as function of position: caudolateral, caudomedial/rostrolateral, and rostromedial neurons corresponded to LF, MF, and HF groups, respectively (**Figure 1**). A total of 162 neurons were recorded, with 50, 57, and 55 cells from the LF, MF, and HF regions, respectively.

### CHARACTERIZATION OF LTK CURRENTS ALONG THE FREQUENCY AXIS OF NL

**FIGURE 2:**
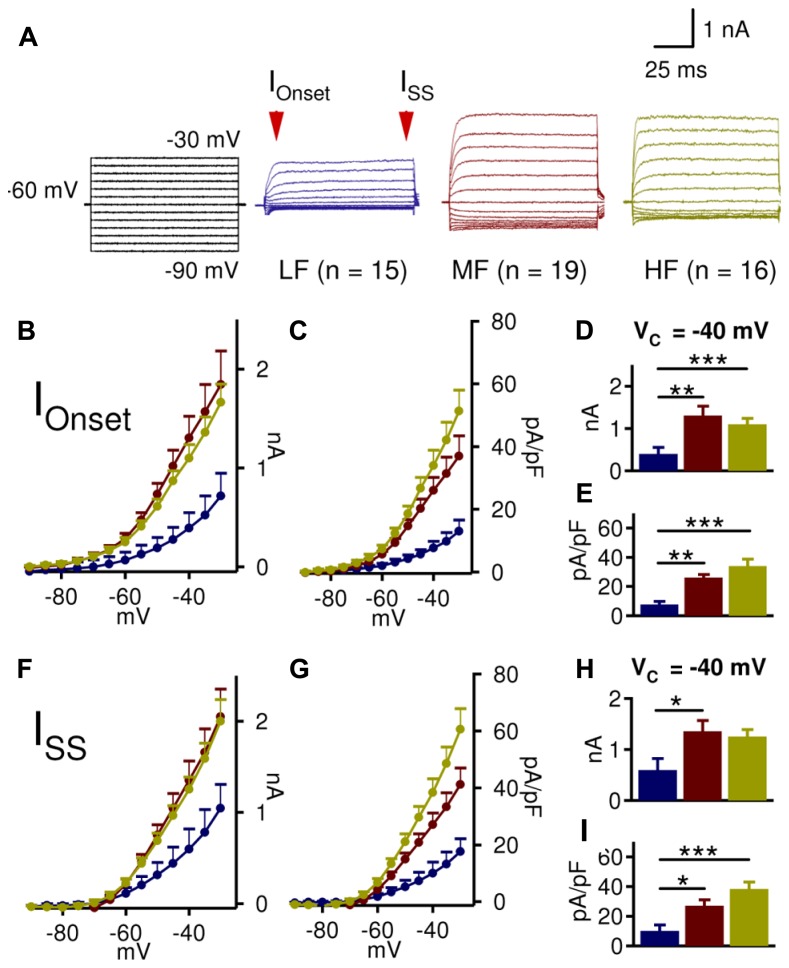
**MF and HF NL neurons have larger low threshold voltage-gated K^+^ (LTK) currents than LF neurons. (A)** Sample protocol (V_hold_ = -60 mV; V_Command_ = -90 to -30 mV, 100 ms duration), and representative LTK current recordings from LF (blue, *n* = 15), MF (red, *n* = 19), and HF (yellow, *n* = 16) NL neurons. **(B,C)** Onset current (I_Onset_) amplitude and current density in MF and HF neurons are higher than those in LF neurons. **(D)** At -40 mV, LF (0.4 ± 0.2 nA) neurons have significantly smaller I_Onset_ than MF (1.3 ± 0.2 nA) and HF (1.1 ± 0.1 nA) neurons. MF and HF neurons do not significantly differ. **(E)** I_Onset_ density at -40 mV shows similar tonotopic variations to I_Onset_ amplitude (LF: 7.6 ± 2.1 pA/pF; MF: 26.1 ± 4.1 pA/pF; and HF: 34.0 ± 4.9 pA/pF). **(F,G)** Steady state current (I_SS_) amplitude and current density reveal similar tonotopic variations to I_Onset_, with higher current amplitude and density in MF and HF neurons compared to LF neurons. **(H)** At -40 mV, LF (0.6 ± 0.2 nA) neurons have significantly smaller I_SS_ than MF (1.4 ± 0.2 nA) and HF (1.3 ± 0.1 nA) neurons. **(I)** I_SS_ density at -40 mV shows similar tonotopic variations to I_SS_ amplitude (LF: 10.0 ± 3.9 pA/pF; MF: 27.1 ± 4.1 pA/pF; and HF: 38.4 ± 4.8 pA/pF). Mean ± SEM are shown in this and subsequent figures. *p < 0.05, ** *p* < 0.01, *** *p* < 0.001 (ANOVA, Tukey’s *post hoc* analysis, unless otherwise indicated). Cells were held at -60 mV for voltage clamp experiments.

In order to assess the interaction between LTK currents and synaptic inhibition, a detailed analysis of LTK currents along the frequency axis of NL was conducted. LTK currents were isolated in the presence of blockers for Na_v_ channels (TTX, 1 μM), low threshold Ca_v_ channels (Mibefrandil, 10 μM), HCN channels (ZD7288, 80 μM), AMPA receptors (DNQX, 20 μM), and GABA_A_ receptors (Gabazine, 20 μM). LTK currents showed a striking tonotopic variation in the amplitude of the onset current (I_Onset_, measured at 4–9 ms after the onset of the V_command_) and steady state current (I_SS_, measured at 94–99 ms) (**Figure 2**). Because K_v_ currents activated at -40 mV primarily represent the LTK component, with little contamination of high threshold K_v_ (HTK) (Brew and Forsythe, 1995; Wang et al., 1998), we analyzed and compared LTK current amplitude at -40 mV. I_Onset_ was significantly smaller in LF (*n* = 15, 0.4 ± 0.2 nA) compared to MF (*n* = 19, 1.3 ± 0.2 nA) and HF (*n* = 16, 1.1 ± 0.2 nA) neurons, and no difference was observed between MF and HF neurons (*p* = 0.002, **Figure 2D**). I_SS_ at -40 mV was also significantly smaller in LF (0.6 ± 0.2 nA) neurons than in MF (1.4 ± 0.2 nA) neurons but not HF (1.3 ± 0.2 nA) neurons (*p* = 0.021, **Figure 2H**). Due to differences in membrane area across the tonotopic axis (gradual reduction in area from LF to MF and HF regions), current density (defined as the ratio of current amplitude over cell capacitance) was compared. Both I_Onset_ and I_SS_ densities at -40 mV were significantly smaller in LF (7.6 ± 4.1 and 10.0 ± 4.5 pA/pF) neurons compared to MF (26.1 ± 3.8 and 27.1 ± 4.0 pA/pF) and HF (34.0 ± 4.0 and 38.4 ± 4.4 pA/pF) neurons (I_Onset_: *p* < 0.0001; I_ss_: *p* < 0.0001, **Figures 2E,I**). These results confirm anatomical data that there is a robust tonotopic variation in LTK channels in NL neurons such that MF and HF neurons have substantially higher LTK current amplitude than LF neurons (Lu et al., 2004; Kuba et al., 2005).

**FIGURE 3:**
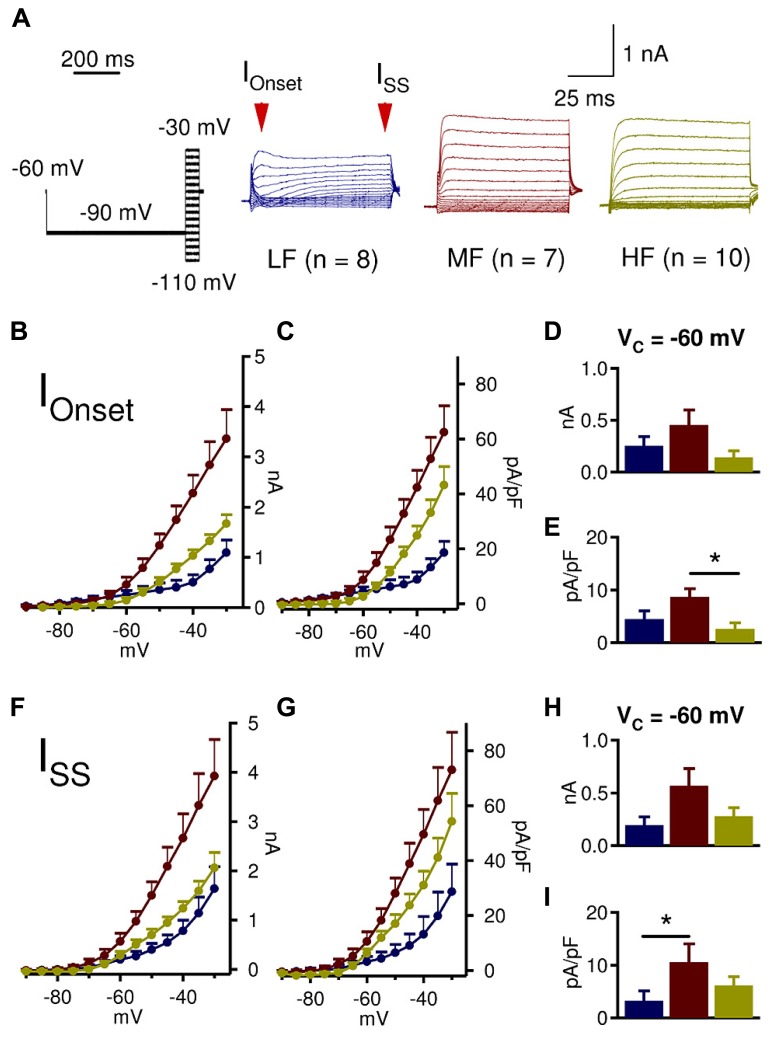
**MF neurons have higher LTK conductance active at rest. (A)** Sample protocol (V_hold_ = -60 mV, V_prepulse_ = -90 mV, 1000 ms duration; V_c_ = -110 to -30 mV, 100 ms duration), and representative LTK current recordings from LF (blue, *n* = 8), MF (red, *n* = 7), and HF (yellow, *n* = 10) NL neurons. **(B–E)** At -60 mV, MF (0.5 ± 0.1 nA) neurons appear to have higher I_Onset_ amplitude than LF (0.3 ± 0.1 nA) and HF (0.2 ± 0.1 nA) neurons. The I_Onset_ density at -60 mV in MF (8.9 ± 1.9 pA/pF) neurons is significantly higher than in HF (2.1 ± 1.7 pA/pF) neurons, while LF neurons have intermediate current density (4.3 ± 1.9 pA/pF). **(F,G)** I_SS_ amplitude and current density reveal similar tonotopic variations to I_Onset_. **(H)** I_SS_ amplitude at -60 mV in MF (0.6 ± 0.1 nA) neurons seems to be higher than LF (0.2 ± 0.1 nA) and HF (0.3 ± 0.1 nA) neurons. **(I)** I_SS_ density at -60 mV in MF (10.9 ± 2.3 pA/pF) neurons is significantly higher than in LF (3.5 ± 2.3 pA/pF) neurons. HF neurons have intermediate current density (5.4 ± 2.0 pA/pF). Kruskal–Wallis tests with follow-up Mann–Whitney *U*-tests. *p < 0.05, **p < 0.01, ***p < 0.001.

Coincident detecting neurons in the auditory brainstem have fast membrane time constants, partly due to a strong active LTK conductance at rest (Kuba et al., 2005; Scott et al., 2005; Mathews et al., 2010). Previous work has suggested that in the NL, MF neurons possess more LTK current at rest than LF and HF neurons (Kuba et al., 2005), however little is known about the amount of active LTK conductances at rest along the tonotopic axis. Therefore, we assessed how much LTK current was available at rest (approximately -60 mV for NL neurons; Reyes et al., 1994; Funabiki et al., 1998; Gao and Lu, 2008) by introducing a -90 mV pre-pulse (1000 ms duration) prior to the voltage commands (100 ms duration) (**Figure 3**). At -60 mV, neither I_Onset_ nor I_SS_ amplitude was significantly different across the tonotopic axis (**Figures 3D,H**). However, the onset and steady state current density of MF neurons were significantly higher than that of LF and HF neurons (**Figures 3E,I**). Specifically, a significant difference in I_Onset_ density between MF (*n* = 7, 8.9 ± 1.9 pA/pF) and HF (*n* = 10, 2.1 ± 1.7 pA/pF) emerged (*p* = 0.038) (**Figure 3E**), and I_SS_ density of MF neurons (10.9 ± 2.3 pA/pF) was higher compared to LF (*n* = 8, 3.5 ± 2.3 pA/pF) neurons (*p* = 0.041, **Figure 3I**). To some extent, LTK current was active at rest in all CF regions, with MF neurons having the largest conductance.

**FIGURE 4:**
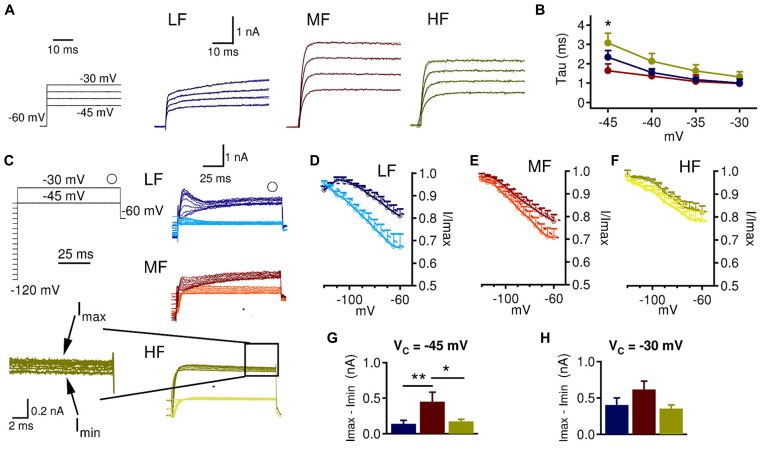
**LTK currents in MF neurons are of faster activation and stronger inactivation than in LF and HF neurons. (A)** To obtain the time constant (τ) for LTK current activation, a single exponential fitting (black dashed line) was performed on the LTK currents recorded at V_c_ from -45 to -30 mV (V_hold_ = -60 mV). **(B)** At -45 mV, MF (1.6 ± 0.3 ms) neurons have significantly faster τ than LF (2.3 ± 0.3 ms) and HF (3.1 ± 0.5 ms) neurons. Kruskal–Wallis tests with follow-up Mann–Whitney *U*-tests. **(C)** Sample protocol used to study LTK inactivation at -45 and -30 mV following increasing voltage steps (-120 to -45 mV, 100 ms duration, and 5 mV increment). The open circle indicates where I_SS_ was measured. The traces with darker colors were obtained at -30 mV and those with lighter colors at -45 mV. Inset shows the I_max_ and I_min_ used to calculate the amount of inactivated current. **(D)** Normalized I_SS_ inactivation at -45 (*n* = 11, V_1/2_ = -83.9 mV; *k* = 19.6) and -30 mV (*n* = 13, V_1/2_ = -71.9 mV; *k* = 7.7) in LF neurons. **(E)** Normalized I_SS_ inactivation at -45 (*n* = 8, V_1/2_ = -83.1 mV; *k* = 14.4) and -30 mV (*n* = 12, V_1/2_ = -82.3 mV; *k* = 13.8) in MF neurons. **(F)** Normalized I_SS_ inactivation at -45 (*n* = 11, V_1/2_ = -85.0 mV; *k* = 13.6) and -30 mV (*n* = 12, V_1/2_ = -82.5 mV; *k* = 8.6) in HF neurons. **(G, H)** The raw amount of inactivated current was calculated by subtracting the maximum current from the minimum I_SS_. MF (0.45 ± 0.8 nA) neurons had significantly more inactivated LTK current at -45 mV than LF (0.14 ± 0.6 nA) and HF (0.19 ± 0.6 nA) neurons. No differences between CF regions were observed in the amount of inactivated LTK current at -30 mV. *p < 0.05, **p < 0.01.

Low threshold voltage-gated K^+^ current kinetics may also vary across the tonotopic axis, which may influence how LTK currents interact with inhibitory synaptic input. Therefore we assessed LTK kinetics across the tonotopic axis by examining LTK activation and steady state inactivation. For activation kinetics, we analyzed the fast membrane time constants (τ < 10 ms) because they are relevant to the peak amplitude and rise time of the depolarizing synaptic conductances. To do this, we used an exponential function, *f*(*t*) = A*exp(-*t*/decay τ ). A fast τ was found in most MF (14/19) and HF (13/16) neurons but in less than half LF (5/14 cells) neurons (**Figure 4A**). The absence of a fast τ in many LF neurons may suggest that LF neurons lack prominent fast activating LTK currents. At -45 mV, MF (1.6 ± 0.3 ms) neurons had a significantly faster τ than LF (2.3 ± 0.3 ms) and HF (3.1 ± 0.5 ms) neurons (**Figure 4B**, *p* = 0.018). LTK inactivation was analyzed from K_v_ currents obtained at two voltages: -45 and -30 mV (**Figure 4C**). While at -45 mV virtually only LTK currents were activated, the current evoked at -30 mV may be contaminated by small amounts of HTK currents (Brew and Forsythe, 1995; Wang et al., 1998). In many recordings, a fast transient inward current was evoked at -45 and -30 mV, likely caused by a low threshold Ca_v_ current incompletely blocked by Mibefrandil (Blackmer et al., 2009). Because the fast inward current corrupted accurate measurement of I_Onset_, we analyzed I_SS_ LTK inactivation. I_SS_ showed relatively little inactivation and similar kinetics across the tonotopic axis. Nonetheless, HF neurons had slightly less LTK channel inactivation (15–25% inactivated) compared to LF (20–30%) and MF (20–30%) neurons (**Figures 4D–F**). MF (*n* = 8, 0.45 ± 0.8 nA) had a larger amount of current inactivation compared to LF (*n* = 11, 0.14 ± 0.6 nA) and HF (*n* = 11, 0.19 ± 0.6 nA) neurons at -45 mV (*p* = 0.008, **Figure 4G**). No tonotopic differences in the amount of current inactivation were observed at -30 mV (**Figure 4H**). Taken together, these data suggest that LTK inactivation kinetics is relatively similar across the tonotopic axis, and the LTK currents are more readily activated in MF neurons.

One caveat to the LTK currents reported here is that the recordings were made at room temperature to achieve a better voltage clamp (Rathouz and Trussell, 1998). LTK currents at physiological temperature would be expected to be slightly larger in peak amplitude and show faster activation kinetics (Cao and Oertel, 2005). Another caveat is the use of embryonic neurons in this study. Although K_v_ currents are relatively mature by E18 (Gao and Lu, 2008), there is evidence that membrane conductances including LTK currents increase after E21 (Kuba et al., 2002). However, the effects of DTX on the firing properties of NL neurons are similar between early chick hatchlings and late embryos (Kuba et al., 2002). Despite these caveats, our data confirm and elaborate on previous research, indicating that LTK current size and kinetics vary across the tonotopic axis of NL.

### CHARACTERIZATION OF IPSPS ALONG THE FREQUENCY AXIS OF NL

**FIGURE 5:**
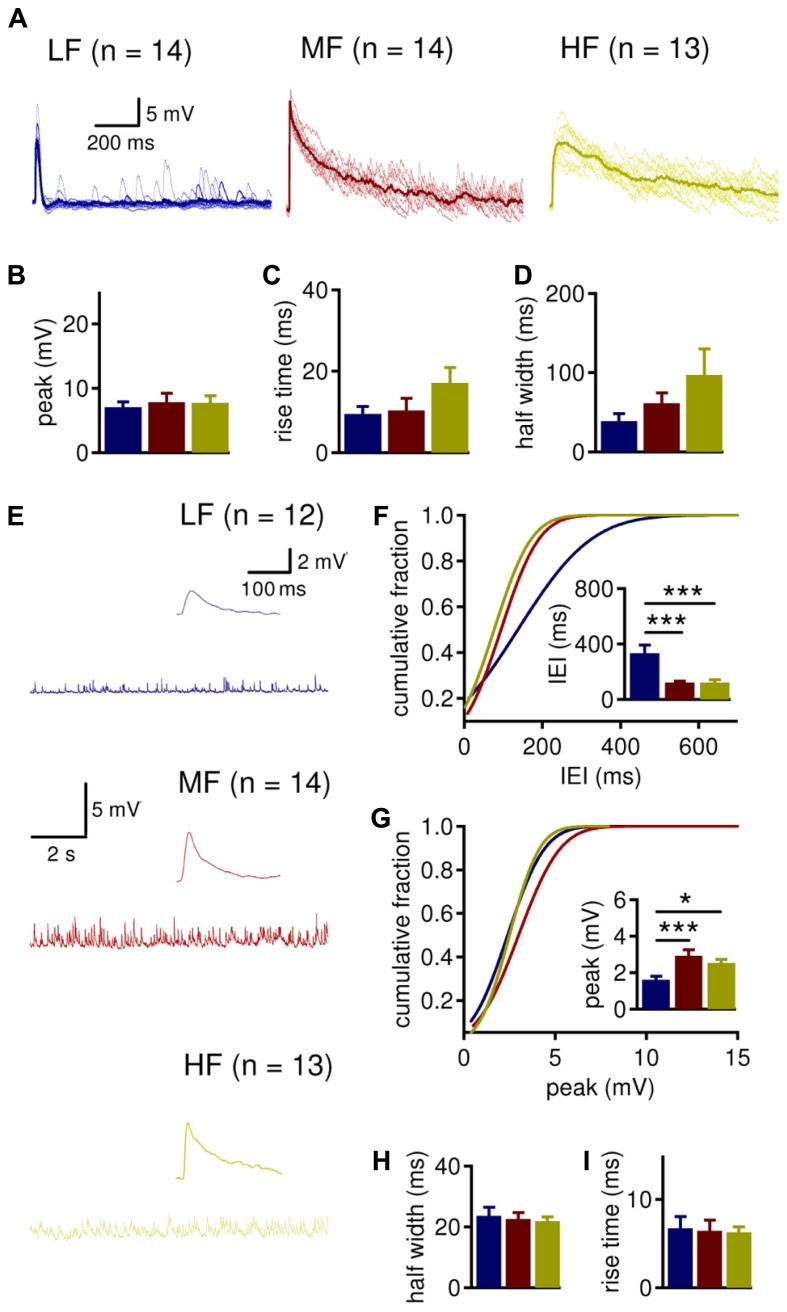
**MF and HF neurons tend to have wider IPSPs and larger sIPSPs than LF neurons. (A)** Sample evoked inhibitory postsynaptic potentials (IPSP) recordings (the average traces shown as thicker lines). A square current pulse (0.2 ms) was delivered to a mixed excitatory and inhibitory fiber tract, and IPSPs were isolated in the presence of DNQX (20 μM). QX-314 (5 mM) was present in the intracellular solution to prevent action potentials in the recorded neurons. LF (blue, *n* = 14) neurons tend to have more narrow IPSPs than MF (red, *n* = 14) and HF (yellow, *n* = 13) neurons. **(B)** Peak IPSP amplitude is not significantly different in LF (7.1 ± 0.8 mV), MF (7.8 ± 1.4 mV), and HF (7.7 ± 1.1 mV) neurons. **(C)** 10–90% rise time of IPSP is not significantly different in LF (9.5 ± 1.9 ms), MF (10.4 ± 3.0 ms), and HF (17.1 ± 3.8 ms) neurons. **(D)** The half width of IPSPs is significantly larger in HF (97.3 ± 32.8 ms) neurons compared to LF (39.0 ± 9.6 ms) neurons. MF (61.3 ± 13.0 ms) neurons do not differ from LF or HF neurons. **(E)** Sample spontaneous IPSP (sIPSP) recordings from LF (blue, *n* = 12), MF (red, *n* = 14), and HF (yellow, *n* = 13) neurons, with a sample single sIPSP shown as an inset. **(F)** MF (120.0 ± 11.0 ms) and HF (120.4 ± 21.4 ms) neurons have significantly shorter inter-event intervals (IEI) than LF (333.3 ± 58.9) neurons. **(G)** LF neurons (1.6 ± 0.2 mV) have smaller sIPSP peak amplitude than MF (3.3 ± 0.3 mV) and HF (2.5 ± 0.2 mV) neurons. **(H,I)** sIPSP half width and 10–90% rise time are not different between CF regions. *p < 0.05, **p < 0.01, ***p < 0.001.

The driving question behind this study is how LTK currents differentially modulate depolarizing inhibitory postsynaptic potentials (IPSPs) in NL neurons. Prior work from our lab has demonstrated a robust tonotopic difference in synaptic inhibition in the NL. LF neurons show less frequent spontaneous inhibitory postsynaptic currents (sIPSCs) than MF and HF neurons, and IPSCs of LF neurons are smaller and faster (Tang et al., 2011; Tang and Lu, 2012). We sought to confirm these tonotopic variations in current clamp recordings (**Figure 5**). Evoked IPSPs were isolated by stimulating the fiber bundle lateral to the NL in the presence of AMPA receptor blocker DNQX (20 μM). To prevent action potentials from occurring on the top of IPSP, QX-314 (5 mM) was included in the intracellular recording solution. A comparison of LTK currents recorded with and without QX-314 in the pipette solution revealed little to no difference in LTK currents (data not shown). No tonotopic differences in IPSP peak amplitude and 10–90% rise time were observed (**Figures 5B,C**). However, IPSP half width was significantly different across the tonotopic axis (**Figure 5D**, *p* = 0.035). The half width of IPSPs was significantly larger in HF (*n* = 13, 97.3 ± 32.8 ms) neurons compared to LF (*n* = 14, 39.0 ± 9.6 ms) neurons, while MF (*n* = 14, 61.3 ± 13.0 ms) neurons had an intermediate value. Analysis of spontaneous IPSPs (sIPSPs) revealed that while there was no tonotopic variation in the sIPSP kinetics (half width and rise time), the frequency and peak amplitude of sIPSPs were different across the tonotopic axis (**Figures 5E–I**). The inter-event interval (IEI) of sIPSPs was significantly smaller in MF (*n* = 14, 120.0 ± 11.0 ms) and HF (*n* = 13, 120.4 ± 21.4 ms) neurons compared to LF (*n* = 12, 333.3 ± 58.9 ms) neurons (**Figure 5F**, *p* = 0.001). Peak amplitude was also significantly different across the tonotopic axis (**Figure 5G**, *p* = 0.003). LF neurons had smaller sIPSP amplitude (1.6 ± 0.2 mV) compared to MF (3.3 ± 0.3 mV) and HF (2.5 ± 0.2 mV). The data on sIPSP frequency and amplitude are consistent with our previous voltage clamp studies on sIPSC parameters (Tang et al., 2011; Tang and Lu, 2012). The lack of differences in sIPSP kinetics across CF regions may result from differential influences of LTK currents and other intrinsic conductances on the depolarizing inhibition.

### INTERACTIONS BETWEEN LTK CURRENTS AND IPSPS ALONG THE FREQUENCY AXIS OF NL

**FIGURE 6:**
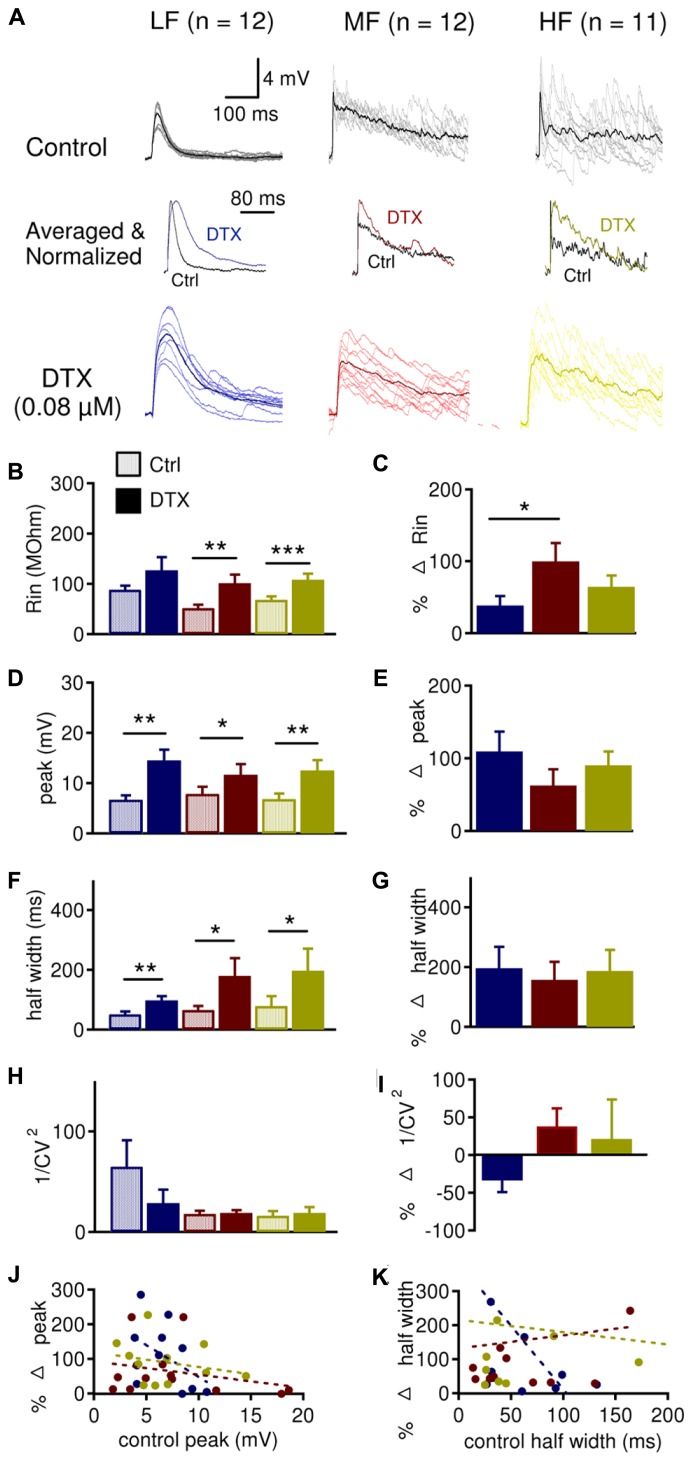
**LTK currents regulate evoked IPSP size in the NL. (A)** Sample IPSP recordings before (dark traces) and after (color traces) bath application of DXT (0.08 μM), in LF (*n* = 12), MF (*n* = 12) and HF (*n* = 11) NL neurons. QX-314 (5 mM) was present in the intracellular solution. Above and below are raw (gray or light color) and averaged (black or dark color) IPSP recordings. Normalized averaged IPSP recordings before (black) and after (color) dendrotoxin I (DTX) are shown in the center. **(B)** DTX significantly increased the input resistance (R_in_) in MF (control: 51.6 ± 6.5 MΩ, DTX: 101.2 ± 16.9 MΩ), and HF (control: 68.6 ± 6.8 MΩ, DTX: 108.1 ± 12.0 MΩ) neurons but not in LF (control: 88.4 ± 8.2 MΩ, DTX: 126.6 ± 26.5 MΩ) neurons. **(C)** R_in_ was increased significantly more in MF (99.8 ± 25.6%) compared to LF (38.3 ± 13.2%) neurons, while HF (64.2 ± 15.8%) did not differ from LF or MF neurons. **(D)** DTX significantly increased the peak IPSP amplitude in LF (control: 6.7 ± 0.8 mV, DTX: 14.5 ± 2.1 mV), MF (control 7.8 ± 1.4 mV, DTX: 11.7 ± 2.1 mV), and HF (control: 6.8 ± 1.1 mV, DTX: 12.5 ± 2.1 mV) neurons. **(E)** No tonotopic differences in the effect of DTX on peak IPSP amplitude were observed. **(F)** DTX significantly increased the half width of IPSP in LF (control: 50.9 ± 9.6 ms, DTX: 97.6 ± 14.8 ms), MF (control: 66.2 ± 13.0 ms, DTX: 179.3 ± 59.2 ms) or HF (control: 79.4 ± 32.8 ms, DTX: 197.4 ± 73.6 ms) neurons. **(G)** No tonotopic differences in the effect of DTX on IPSP half width. **(H)** 1/CV^2^was not significantly changed in LF, MF, or HF neurons after DTX application. **(I)** Percent change in 1/CV^2^was not significantly different across the tonotopic axis. **(J)** Peak IPSP amplitude plotted against the percent change in peak amplitude after DTX reveals that neurons with smaller IPSP peak amplitudes showed a greater change in IPSP peak amplitude after DTX treatment. The change appeared to be larger in LF (*r*^2^ = 0.215, *p* = 0.076) neurons than MF (*r*^2^ = 0.067) and HF (*r*^2^ = 0.061) neurons. **(K)** IPSP half width plotted against the percent change in half width after DTX reveals that neurons with smaller IPSP half width showed a greater change in IPSP half width after DTX treatment. The change appeared to be larger in LF (*r*^2^ = 0.347, *p* = 0.022) neurons than MF (*r*^2^ = 0.009) and HF (*r*^2^ = 0.010) neurons. *p < 0.05, **p < 0.01, ***p < 0.001.

**FIGURE 7:**
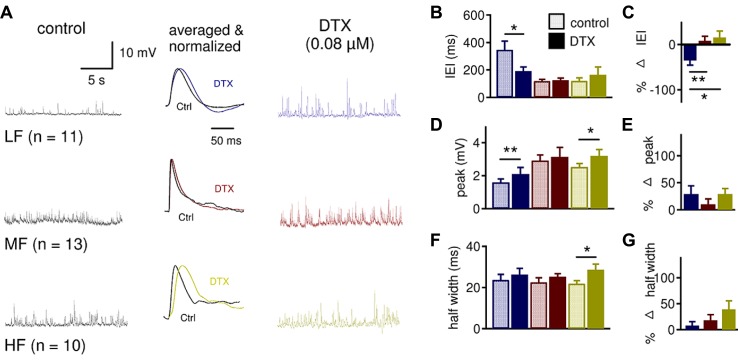
**LTK currents strongly regulate sIPSP frequency in LF neurons and sIPSP shape in HF neurons. (A)** Sample sIPSP recordings before (dark traces) and after (color traces) bath application of DTX (0.08 μM) in LF (*n* = 11), MF (*n* = 13) and HF (*n* = 10) NL neurons. See **Figure 6A** for legend. **(B)** The IEI significantly decreased in LF (control: 347.2 ± 63.1 ms, DTX: 192.3 ± 30.2 ms) but not MF (control: 120.0 ± 11.0 ms, DTX: 119.4 ± 12.8 ms) and HF (control: 129.4 ± 24.8 ms, DTX: 164.5 ± 57.2 ms) neurons after DTX. **(C)** The percent decrease in IEI of sIPSPs is significantly greater in LF (-35.3 ± 10.4%) neurons compared to MF (8.1 ± 9.8%) and HF (15.5 ± 14.4%) neurons. **(D)** There is a significant increase in sIPSP peak amplitude after DTX treatment in LF (control: 1.5 ± 0.2 mV, DTX: 2.1 ± 0.4 mV) and HF (control: 2.5 ± 0.2 mV, DTX: 3.2 ± 0.4 mV) neurons but not in MF (control: 3.0 ± 0.3 mV, DTX: 3.1 ± 0.6 mV) neurons. **(E)** The percent change in sIPSP peak amplitude was not different across the tonotopic axis. **(F)** DTX increased the sIPSP half width in HF (control: 21.9 ± 1.7 ms, DTX: 28.6 ± 2.8 ms) but not in LF (control: 23.7 ± 2.7 ms, DTX: 26.3 ± 3.0 ms) and MF (control: 22.5 ± 2.1 ms, DTX: 25.2 ± 1.5 ms) neurons. **(G)** Although HF neurons show a substantial increase in sIPSP half width, the percent change in sIPSP half width is not significantly different across the tonotopic axis (*p* = 0.075). *p < 0.05, **p < 0.01, ***p < 0.001.

To assess the role of LTK currents in regulating subthreshold changes in membrane potentials caused by activation of inhibitory inputs to NL neurons, we studied the effects of DTX (0.08 μM), a selective blocker for K_v1_-subunit containing channels (Harvey, 2001), on IPSPs (**Figure 6**) and sIPSPs (**Figure 7**). DTX significantly increased the input resistance (R_in_) in MF (*n* = 12, control: 51.6 ± 6.5 MΩ, DTX: 101.2 ± 16.9 MΩ, *p* = 0.008) and HF (*n* = 11, control: 68.6 ± 6.8 MΩ, DTX: 108.1 ± 12.0 MΩ, *p* = 0.001) neurons, but not in LF (*n* = 12, control: 88.4 ± 8.2 MΩ, DTX: 126.6 ± 26.5 MΩ, *p* = 0.054) neurons (**Figure 6B**). R_in_ was increased significantly more in MF (99.8 ± 25.6%) compared to LF (38.3 ± 13.2%) neurons (*p* = 0.028) (**Figure 6C**), agreeing with Kuba et al. (2005). DTX significantly increased the peak IPSP amplitude in all CF regions: LF (control: 6.7 ± 0.8 mV, DTX: 14.5 ± 2.1 mV, *p* = 0.004), MF (control: 7.8 ± 1.4 mV, DTX: 11.7 ± 2.1 mV, *p* = 0.030), and HF (control: 6.8 ± 1.1 mV, DTX: 12.5 ± 2.1 mV, *p* = 0.002, **Figure 6D**). DTX also increased the half width of IPSPs all CF regions: LF (control: 50.9 ± 9.6 ms, DTX: 97.6 ± 14.8 ms, *p* = 0.002), MF (control: 66.2 ± 13.0 ms, DTX: 179.3 ± 59.2 ms, *p* = 0.040) and HF (control: 79.4 ± 32.8 ms, DTX: 197.4 ± 73.6 ms, *p* = 0.028) neurons (**Figure 6F**). No tonotopic differences in the percent effect of DTX on IPSP peak amplitude or half width were detected (**Figures 6E,G**). The analysis of coefficient of variation (CV) of synaptic responses can be used as one indicator of whether changes in IPSP size and shape is due to pre- or postsynaptic mechanism (Scheuss et al., 2002; Clements, 2003). No significant differences in 1/CV^2^ were found between control and DTX conditions, nor among CF regions (**Figures 6H–I**). However, LF (64.9 ± 26.1) neurons had significantly larger 1/CV^2^ in control conditions than MF (18.0 ± 3.2) and HF (16.1 ± 4.6) neurons (*p* = 0.041, not shown in figure) and LF neurons tended to show a reduction in 1/CV^2^ after DTX application (control: 64.9 ± 26.1; DTX: 28.7 ± 13.5; *p* = 0.069). LF neurons also showed a correlation between control IPSP peak amplitude and half width with their respective percent changes (amplitude: *r*^2^ = 0.215, *p* = 0.076; half width: *r*^2^ = 0.347, *p* = 0.022) after DTX application (**Figures 6J,K**). These data demonstrate that LTK currents regulate the size and shape of IPSPs in NL neurons across the entire frequency axis and suggest the possibility of a presynaptic component of this effect in LF neurons.

These findings were somewhat surprising because MF and HF neurons had stronger LTK current amplitude and density than LF neurons (**Figures 2 and 3**), and therefore MF and HF neurons were expected to show a greater change in IPSP parameters than LF neurons. One potential explanation for this apparent discrepancy is that presynaptic LTK currents regulate the inhibitory inputs to LF neurons to a greater extent than in MF and HF neurons. Analysis of the effects of DTX on sIPSP supported this hypothesis (**Figure 7**). DTX significantly decreased IEI in LF (*n* = 11, control: 347.2 ± 63.1 ms, DTX: 192.3 ± 30.2 ms, *p* = 0.011) but not in MF (*n* = 13, control: 119.4 ± 12.8 ms, DTX: 124.7 ± 15.1 ms, *p* = 0.646) and HF (*n* = 10, control: 129.4 ± 24.8 ms, DTX: 164.5 ± 57.2 ms, *p* = 0.374) neurons (**Figure 7B**). The percent decrease in IEI was significantly different in LF (-35.3 ± 10.4%) neurons compared to MF (8.1 ± 9.8%) and HF (15.5 ± 14.4%) neurons (*p* = 0.017, **Figure 7C**). There was a significant increase in sIPSP peak amplitude after DTX treatment in LF (control: 1.5 ± 0.2 mV, DTX: 2.1 ± 0.4 mV, *p* = 0.010) and HF (control: 2.5 ± 0.2 mV, DTX: 3.2 ± 0.4 mV, *p* = 0.018) neurons but not in MF neurons (**Figure 7D**). DTX increased in sIPSP half width in HF (control: 21.9 ± 1.7 ms, DTX: 28.6 ± 2.8 ms, *p* = 0.038) but not LF and MF neurons (**Figure 7G**). In spite of robust tonotopic differences in postsynaptic LTK currents, there were no significant differences in the effect of DTX on percent changes in peak amplitude or half width of sIPSP among LF, MF and HF neurons (**Figures 7E,G**), and LTK currents appear to regulate the sIPSP frequency in LF neurons, suggesting that presynaptic LTK currents regulate inhibitory synapses.

### LTK CURRENTS ON IPSCs: ACTION LOCI OF DTX

**FIGURE 8:**
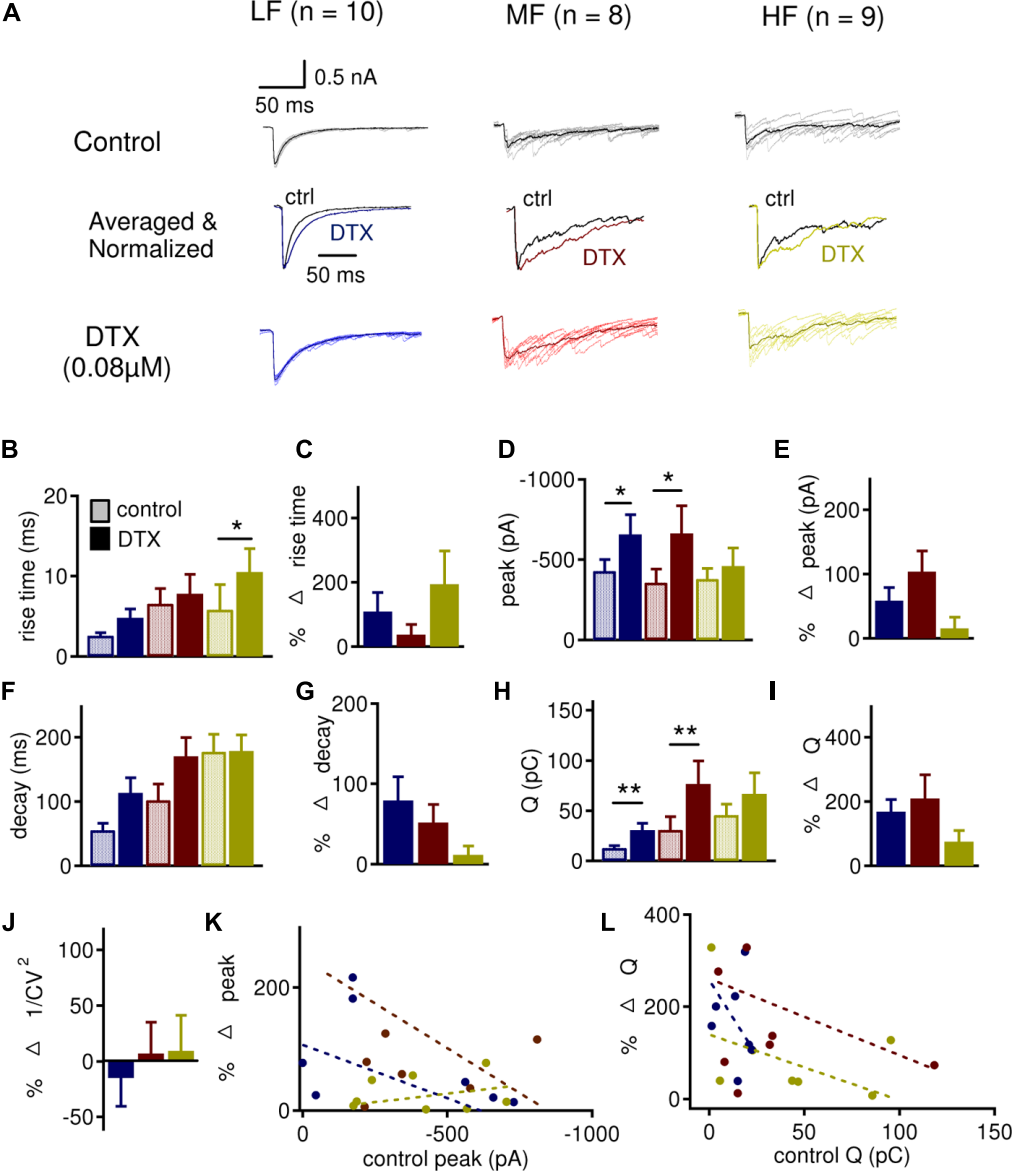
**DTX preferentially regulates IPSC frequency and shape in LF and MF neurons. (A)** Sample evoked IPSC recordings before (dark traces) and after (color traces) bath application of DXT (0.08 μM) in LF (*n* = 10), MF (*n* = 8), and HF (*n* = 9) NL neurons. See **Figure 6A** for legend. **(B,C)** DTX increases the 10–90% rise time in HF (control: 5.8 ± 3.3 ms, DTX: 10.5 ± 3.1 ms) neurons, but not in LF (control: 2.6 ± 0.4 ms, DTX: 4.8 ± 1.2 ms) and MF (control: 6.1 ± 1.9 ms, DTX: 6.6 ± 2.4 ms) neurons, without significant differences in percent changes across tonotopic axis. **(D)** DTX increased the IPSC peak amplitude in LF (control: -429.8 ± 72.6 pA, DTX: -656.4 ± 112.7 pA) and MF (control: -357.1 ± 83.3 pA, DTX: -662.5 ± 172.7 pA) but not in HF (control: -379.9 ± 66.6 pA, DTX: -460.4 ± 113.4 pA) neurons. **(E)** Percent change in IPSC peak amplitude is not significantly different between LF (58.5 ± 20.6%), MF (103.8 ± 32.5%), and HF (15.2 ± 17.4%) neurons. **(F)** No change in the decay time constant was observed in LF (control: 55.4 ± 10.7 ms, DTX: 113.5 ± 23.7 ms), MF (control: 101.9 ± 25.3 ms, DTX: 170.5 ± 29.3 ms) or HF (control: 158.8 ± 27.3 ms, DTX: 178.7 ± 25.1 ms) neurons. **(G)** Percent change in IPSC decay time constant is not significantly different across CF regions. **(H)** DTX increases the amount of charge per IPSC (Q) in LF (control: 12.7 ± 2.4 pC, DTX: 30.7 ± 7.3 pC) and MF (control: 31.0 ± 13.0 pC, DTX: 76.4 ± 22.5 pC) neurons but not in HF (control: 45.6 ± 10.8 pC, DTX: 66.6 ± 20.9 pC) neurons. **(I)** The percent change in Q is not different between CF regions. **(J)** No significant differences are observed between CF regions in 1/CV^2^ after DTX treatment. **(K,L)** Control peak amplitude and total area plotted against the percent change in peak amplitude and total area, respectively, after DTX reveals that there was little to no correlation between control and percent change after DTX in all CF regions: LF (peak amplitude: *r*^2^ = 0.076; area: *r*^2^ = 0.156), MF (amplitude: *r*^2^ = 0.140; area: *r*^2^ = 0.088), and HF (amplitude: *r*^2^ = 0.060; area: *r*^2^ = 0.190). *p < 0.05, **p < 0.01.

To better understand the presynaptic versus postsynaptic roles of LTK currents in regulating synaptic inhibition, we studied the effects of DTX on IPSCs recorded under voltage clamp (V_h_ = -60 mV). Ideally, under voltage clamp, changes in IPSCs observed after DTX application should be attributable to blockade of presynaptic LTK channels because postsynaptic LTK channels are not activated. Evoked IPSCs were subject to LTK modulation (**Figure 8**). DTX tended to increase the peak amplitude, decay time constant, and amount of charge (Q) in LF neurons (*n* = 10; peak amplitude: control: -429.8 ± 72.6 pA, DTX: -656.4 ± 112.7 pA, *p* = 0.031; decay time constant: control 55.4 ± 10.7 ms, DTX: 113.5 ± 23.7 ms, *p* = 0.071; Q: control: 12.7 ± 2.4 pC, DTX: 30.7 ± 7.3 pC, *p* = 0.012), and in MF neurons (*n* = 8; peak amplitude: control: -357.1 ± 83.3 pA, DTX: -662.5 ± 172.7 pA, *p* = 0.018; decay time constant: control: 101.9 ± 25.3 ms, DTX: 170.5 ± 29.3 ms, *p* = 0.063; Q: control: 31.0 ± 13.0 pC, DTX: 76.4 ± 22.5 pC, *p* = 0.012) (**Figures 8D,F,H**). HF neurons (*n* = 9) did not show increases in peak amplitude, decay time constant, and amount of charge, but showed a significant increase in 10–90% rise time (control 5.8 ± 3.3 ms, DTX: 10.5 ± 3.1 ms, *p* = 0.045) (**Figure 8B**). The percent change in 10–90% rise time, peak, decay time constant, and amount of charge did not differ across the tonotopic axis (**Figures 8C,E,G,I**). These data suggest that presynaptic LTK may, to some extent, regulate IPSCs in all CF regions but more prominently in LF neurons.

**FIGURE 9:**
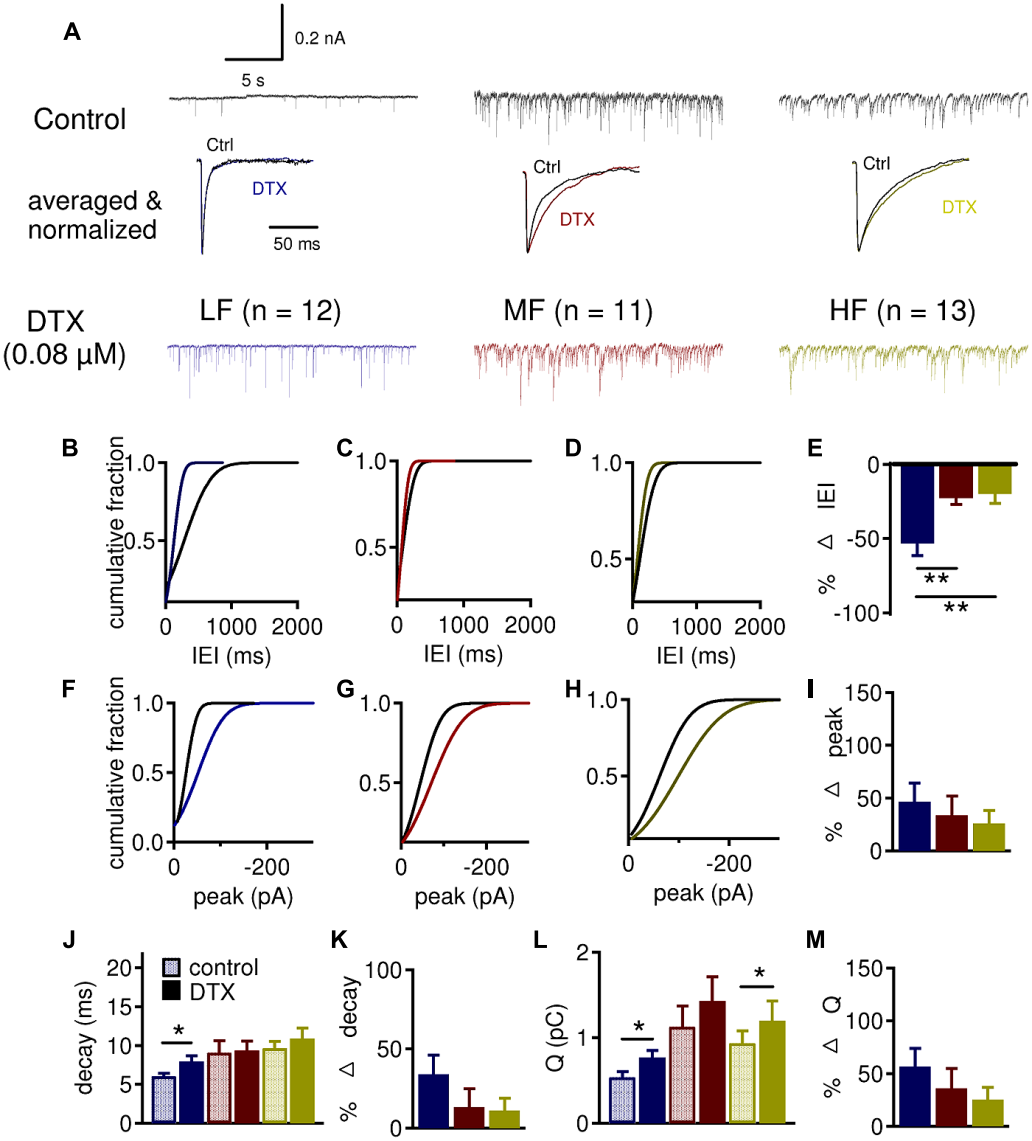
**LTK currents strongly regulate the frequency of sIPSCs in LF neurons. (A)** Sample sIPSC recordings before (dark traces) and after (color traces) bath application of DXT (0.08 μM) in LF (blue, *n* = 12), MF (red, *n* = 11) and HF (yellow, *n* = 13) NL neurons. See **Figure 6A** for legend. **(B–D)** Cumulative fraction of IEI in LF **(B)**, MF **(C)**, and HF **(D)** neurons before (black) and after (color) DTX. **(E)** Percent change in IEI is significantly larger in LF (-53.1 ± 8.3%) neurons compared to MF (-22.7 ± 4.2%) and HF (-19.8 ± 6.4%) neurons. No differences were observed between MF and HF neurons. **(F–H)** Cumulative fraction of sIPSC IEI in LF **(F)**, MF **(G)**, and HF **(H)** neurons before (black) and after (color) DTX. **(I)** No significant differences in percent change in sIPSC peak after DTX treatment between LF (46.6 ± 17.7%), MF (33.7 ± 18.2%), and HF (26.1 ± 12.2%) neurons. **(J)** The sIPSC decay time constant is significantly increased in LF (control: 6.0 ± 0.4 ms, DTX: 8.0 ± 0.7 ms) but not in MF (control: 9.1 ± 1.5 ms, DTX: 9.3 ± 1.2 ms) or HF (control: 9.7 ± 0.8 ms, DTX: 10.9 ± 1.4 ms) neurons. **(K)** No significant differences in percent change in the sIPSC decay time constant were observed between LF, MF, and HF neurons after DTX treatment. **(L)** The amount of charge transferred per sIPSC (Q) is significantly increased in LF (control: 0.5 ± 0.1 pC, DTX: 0.8 ± 0.1 pC) and HF (control: 0.9 ± 0.1 pC, DTX: 1.2 ± 0.2 pC) but not in MF (control: 1.1 ± 0.2 pC, DTX: 1.4 ± 0.3 pC) neurons. **(M)** No significant differences in percent change in sIPSC area after DTX treatment across CF regions. *p < 0.05, **p < 0.01.

To further confirm and define the role of presynaptic LTK currents, we studied the effect of DTX on spontaneous IPSCs (sIPSCs; **Figure 9**). Cumulative probability of sIPSC IEI revealed that DTX decreased IEI predominantly in LF (*n* = 12, -53.1 ± 8.3%) neurons compared to MF (*n* = 11, -22.7 ± 4.2%) and HF (*n* = 13, -19.8 ± 6.4%) neurons (**Figures 9B–E**, *p* = 0.002). sIPSC peak amplitude also similarly increased after DTX application in LF (46.6 ± 17.7%), MF (33.7 ± 18.2%), and HF (26.1 ± 12.2%) neurons (**Figures 9F–I**). sIPSC decay time constant was significantly increased in LF (control: 6.0 ± 0.4 ms, DTX: 8.0 ± 0.7 ms, *p* = 0.022) but not in MF (control: 9.1 ± 1.5 ms, DTX: 9.3 ± 1.2 ms, *p* = 0.829) or HF (control: 9.7 ± 0.8 ms, DTX: 10.9 ± 1.4 ms, *p* = 0.193) neurons (**Figure 9J**). The amount of charge transferred per sIPSC (Q) increased after DTX application in LF (control: 0.5 ± 0.1 pC, DTX: 0.8 ± 0.1 pC, *p* = 0.011) and HF (control: 0.9 ± 0.1 pC, DTX: 1.2 ± 0.2 pC, *p* = 0.040), but not in MF (control: 1.1 ± 0.2 pC, DTX: 1.4 ± 0.3 pC, *p* = 0.078) neurons (**Figure 9L**). No differences in the percent change in peak amplitude, decay time constant, and charge were observed between LF, MF, and HF neurons (**Figures 9I,K,M**). These data support the notion that LTK currents have a presynaptic role in modulating IPSC size and shape in NL neurons especially in LF neurons. The discrepancy in the effects of DTX on sIPSCs versus evoked IPSCs in MF neurons might be caused by relatively weak influences of LTK currents on the spontaneous release of GABA in MF neurons.

### LTK CURRENTS ON EXCITABILITY OF NL NEURONS

**FIGURE 10:**
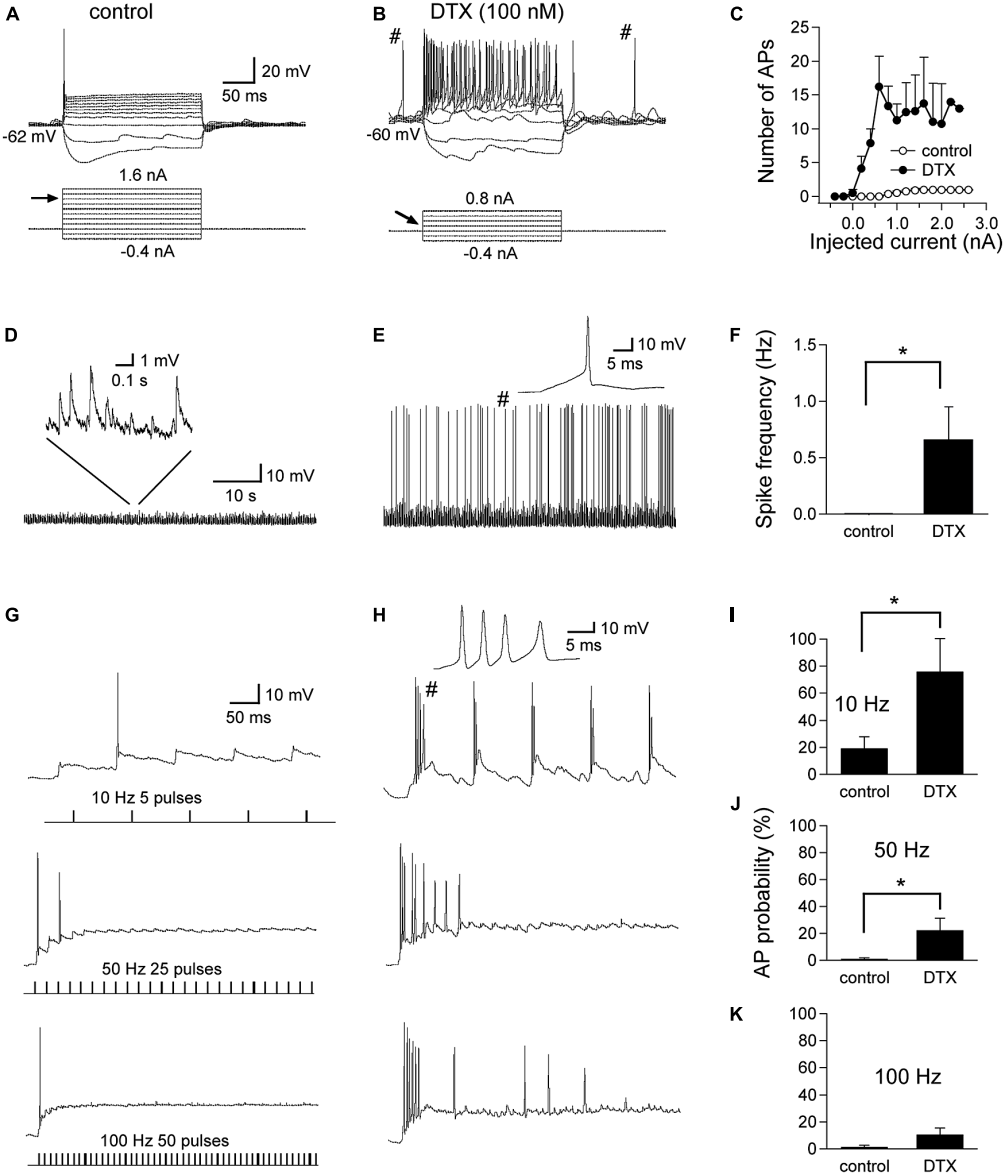
**Regulation of GABA responses in NL neurons by LTK channels. (A)** Under control conditions, the cell fires one single action potential (AP) in response to prolonged suprathreshold current injections (threshold current indicated by the arrow, 1.2 nA in this case). **(B)** DTX slightly depolarizes the RMP, substantially lowers the threshold current to 0.2 nA, and changed the phasic firing pattern to a tonic mode. **(C)** Pooled data show a dramatic increase in the number of APs in response to DTX application (*n* = 9). Furthermore, spontaneous APs, which are absent under control condition, appear prior to and after the current injections (indicated by #). Because ionotropic glutamate receptors were blocked throughout the experiments, these two spontaneous spikes likely occurred on top of two depolarizing IPSPs. **(D)** Chart recordings show sIPSP with amplitude of up to several millivolts, without spike activity. **(E)** DTX increased the amplitude of sIPSP and transforms some into spikes (one spike indicated by # is shown at an enlarged time scale). **(F)** Spike frequency was significantly higher after DTX application than in control conditions (*n* = 9). **(G,H)** Train stimulations at different frequencies were applied to evoke GABA responses. At 50 and 100 Hz, IPSPs summate, forming a sustained membrane depolarization of about 15 mV. GABA-induced spikes were seen occasionally. Under DTX, more spikes were seen at all three frequencies tested, and bursts of spikes are noted (a burst of four APs shown at an enlarged time scale). **(I–K)** Significant increase in AP probability was detected for the stimulations at frequency of 10 and 50 Hz but not 100 Hz (*n* = 9). *p < 0.05.

Finally, we assessed the role of LTK currents in regulating neuronal excitability in NL. Specifically, we tested whether the presence of LTK currents prevented GABA-induced action potentials in NL neurons, as suggested in NM neurons (Monsivais et al., 2000; Howard et al., 2007). We thus investigated such interactions in NL neurons using current clamp recordings (**Figure 10**). We first confirmed the effects of DTX (0.1 μM) on the intrinsic firing properties of NL neurons in MF and HF regions. Under control conditions, NL cells fired one action potential in response to prolonged suprathreshold current injections followed by a plateau of subthreshold membrane potential (**Figure 10A**), a characteristic hallmark of time-coding neurons in the central auditory system. DTX produced a small depolarization (2 mV) in RMP, substantially lowered the threshold current, and changed the phasic firing pattern to a tonic mode (**Figures 10B,C**), consistent with previous findings in auditory brainstem neurons where K_v1_-containing channels are highly expressed (e.g., Brew and Forsythe, 1995; Gittelman and Tempel, 2006). Furthermore, spontaneous action potentials, which were absent under control conditions, appeared prior to the onset and after the termination of the current injections. Because ionotropic glutamate receptors were blocked throughout these experiments, the spontaneous spikes occurred on the top of depolarizing IPSPs. Under control condition, sIPSP varied widely in amplitude, with maximal membrane depolarization of up to several millivolt without spike activity (**Figure 10D**). DTX increased the amplitude of sIPSP and transformed some into spikes (**Figures 10E,F**, *n* = 9). To further study the effects of DTX on evoked IPSP and GABA-induced spikes, train stimulations at different frequencies were applied to evoke GABA responses. At 50 and 100 Hz, IPSPs summated temporally, forming a sustained membrane depolarization of about 15 mV. GABA-induced spikes were seen occasionally (**Figure 10G**). Under DTX, more spikes were seen at all three frequencies tested, and bursts of spikes occurred at the beginning of the simulation. Significant increase in AP probability was detected for the stimulations at frequency of 10 and 50 Hz (**Figures 10H,I**, *n* = 9). These data confirm that LTK currents prevent GABA-induced excitation, providing a critical role for LTK currents in maintaining synaptic inhibition in NL.

## DISCUSSION

This study sought to determine the interplay between intrinsic LTK currents and extrinsic synaptic inhibition in NL neurons across the tonotopic axis. We characterized the LTK currents in LF, MF, and HF neurons, and then demonstrated a tonotopic relationship between LTK currents and inhibitory synaptic input. Interestingly, the data suggest that while MF and HF neurons possess larger postsynaptic LTK currents than LF neurons, robust presynaptic LTK currents in LF neurons may compensate for the relatively lower postsynaptic LTK counterpart, leading to equally strong LTK influences on synaptic inhibition across different frequency-coding regions. In addition to shaping inhibitory inputs, LTK currents also prevent GABAergic-driven excitation and therefore are critical for the maintenance of inhibition in NL.

Postsynaptic LTK currents in NL neurons exhibit tonotopic variation in amplitude and kinetics along the tonotopic axis of NL. LTK currents were substantially higher in MF and HF neurons compared to LF neurons. The presence of a tonotopic gradient of K_v_ currents, with stronger current expression with increasing CF, has been shown in peripheral (Pantelias et al., 2001) and a variety of central auditory structures (Li et al., 2001; Fukui and Ohmori, 2004; Brew and Forsythe, 2005; Kuba et al., 2005). In NL, the LTK currents seem to be most specialized in MF neurons. One important measure of the LTK channels in timing coding neurons in the auditory system is the amount of active current at rest. It is well known that active LTK conductances at rest contribute to making the membrane leaky, and result in a narrow time window for converging excitatory inputs to drive the cells to spike (Golding et al., 1999; Scott et al., 2005). Although all NL neurons possessed active LTK conductance at rest, MF neurons had a higher LTK current density at rest than LF and HF neurons (**Figures 3 and 6**), consistent with the observation of specialized fast membrane time constants and fast synaptic inputs in MF neurons (Kuba et al., 2005). Furthermore, in terms of activation kinetics of LTK currents, MF neurons had the fastest τ at -45 mV compared to LF and HF neurons. The activation kinetics of LTK currents in MF neurons indeed were similarly fast compared to those found in NM neurons (Rathouz and Trussell, 1998), with τ ranging from 2 to 1 ms at membrane potentials between -45 and -30 mV. These data reaffirm that MF neurons have a more active LTK conductance at low membrane potentials. There were, however, no significant differences in inactivation kinetics of LTK currents across the tonotopic axis. A relatively small portion (~20%) of steady state LTK currents in NL neurons inactivate at -45 to -30 mV, similar to NM neurons (Rathouz and Trussell, 1998).

One potential caveat in these experiments would be errors in current measurement due to poor space clamping, particularly in LF neurons, which are known to have longer dendrites than MF and HF neurons. While long dendrites can introduce space clamp errors, the number of dendritic bifurcations and the diameter of primary dendrites can also dramatically influence space clamp. Specifically, thin primary dendrites with numerous bifurcations will also introduce relatively large space clamp errors. Furthermore, the membrane resistance is also a major factor in determining the degree of space clamp errors (Bar-Yehuda and Korngreen, 2008; Poleg-Polsky and Diamond, 2011). Space clamp errors are unlikely to account for the differences in LTK amplitude across the tonotopic axis, because the dendritic gradient in the NL is such that LF neurons have longer, thicker primary dendrites with relatively few bifurcations. With increasing CF, dendritic length and diameter decrease, while the number of primary dendrites and bifurcations increase (Smith and Rubel, 1979). Additionally, MF neurons have lower R_in_ than LF and HF neurons (Kuba et al., 2005). Therefore, given the tonotopic differences in R_in_, dendritic length, width and branching, we expect that space clamp errors did not contribute to the significant differences in LTK currents across the tonotopic axis.

Given the tonotopic arrangement of LTK currents in NL, we expected to find more robust change in IPSP shape and size in MF and HF neurons than in LF neurons after application of K_v1_ channel blocker DTX. To the contrary, the effect of DTX was about equally evident in all CF regions. We found increases in IPSP and sIPSP peak amplitude and half width after DTX treatment in most CF regions. Although MF neurons have more LTK currents active at rest, there were no significant differences in the percent changes caused by DTX in the size and shape of IPSP and sIPSP between LF, MF, and HF neurons. The concurrent increase in sIPSP frequency (reduction in sIPSP IEI) in LF but not MF and HF neurons suggests that presynaptic LTK currents may have contributed to the changes in IPSP amplitude in LF neurons. We tested this hypothesis by conducting voltage clamp experiments, which should minimize the influence of postsynaptic LTK currents and therefore allow us to assess to what extent presynaptic LTK currents contributed to DTX-induced changes in the size and shape of the synaptic inhibitory responses. Our data were suggestive of a tonotopic arrangement of presynaptic LTK currents in NL, which is supported by the observation that DTX induced an increase in sIPSC frequency preferentially in LF neurons. Furthermore, DTX caused significant changes in the peak, decay, and amount of charge (Q) of IPSCs and sIPSCs in LF neurons, reflecting a combined effect of both presynaptic and postsynaptic LTK currents on the synaptic inhibition in LF neurons. Fewer parameters of IPSC were affected by DTX in MF neurons, and the effects of DTX on IPSCs in HF neurons were even less significant. These results suggest that presynaptic LTK currents are more prevalent in LF than in MF and HF neurons. A recent study (Yamada et al., 2013) demonstrates that local GABAergic neurons project primarily to LF neurons. It remains to be determined whether these GABAergic terminals express stronger LTK channels than those that originate from the SON.

Presynaptic mechanisms can be confirmed with analysis of variability (1/CV^2^) of peak amplitude of evoked synaptic responses and paired pulse ratio (PPR; del Castillo and Katz, 1954; Oleskevich et al., 2000; Scheuss et al., 2002). PPR paradigm, however, is not effective in studying synaptic inhibition of NL neurons due to dramatic fluctuations in IPSC peak amplitude under control conditions (Kuo et al., 2009; Tang et al., 2013). Analyses of 1/CV^2^ of IPSPs (**Figures 6H,I**) and IPSCs (**Figure 8J**) did not show significant differences in the percent change of 1/CV^2^ caused by DTX between CF regions. However, DTX tended to substantially reduce 1/CV^2^ in LF neurons while slightly increasing 1/CV^2^ in MF and HF neurons (**Figures 6 and 8**), suggesting that presynaptic LTK currents preferentially influence inhibitory synaptic input in LF neurons. We speculate that there may be a tonotopic arrangement of presynaptic LTK currents opposing the postsynaptic arrangement of LTK currents. In other words, postsynaptic LTK currents are largest in HF neurons while presynaptic LTK currents are largest in LF neurons. To support this hypothesis, further research will need to demonstrate the presence of LTK-subfamily channels (e.g.; K_v1_, K_v4_, K_v7_) on inhibitory synapses in the NL.

The presence of presynaptic LTK channels on inhibitory terminals of LF neurons provides an intriguing possibility for interplay between these two neuronal properties that may be critical for forming fast inhibition in LF neurons. The fast kinetics of IPSCs in LF NL neurons can be attributed to both a presynaptic release profile with strong synchronization (Tang and Lu, 2012) and a postsynaptic enrichment of the fast α1-GABA_A_receptor subunit (Yamada et al., 2013). MF and HF neurons display stronger asynchronous release of GABA than LF neurons (Tang and Lu, 2012), which is consistent with the results of the current study (**Figures 5, 6, and 8**). We propose that in addition to these mechanisms, presynaptic LTK currents also contribute to accelerating IPSCs in LF neurons. In fact, K_v1_-containing channels have been shown to regulate presynaptic spiking activity in a variety of structures, including the cerebellum, hippocampus, and motor nerve (Trussell and Roberts, 2008). After DTX application, a fair amount of asynchronous events emerged in LF neurons, whereas qualitatively there was not an obvious change in MF and HF neurons (**Figures 6 and 8**), supporting a role of presynaptic LTK channels in LF neurons. This suggests that LF neurons may utilize similar mechanisms including intrinsic voltage-gated conductances and fast synaptic inhibition to code ITD as observed in mammalian medial superior olive neurons (Grothe and Sanes, 1993, 1994; Brand et al., 2002; Dodson et al., 2002, 2003; Grothe, 2003; Roberts et al., 2013). While presynaptic LTK currents may regulate fast phasic inhibition in LF neurons, postsynaptic LTK currents prevent GABA-induced excitation. In NM neurons, GABA-driven excitation is prevalent in E14 chicks, but decreases during development and becomes predominantly inhibitory at E18. The decrease in GABA-induced excitation coincides with the increase in LTK currents (Howard et al., 2007). The same principle may apply to the maturation of GABAergic inhibition in NL neurons. The LTK currents are thus critical not only in switching the sign of GABA inputs from excitation to inhibition but also maintenance of synaptic inhibition in coincidence detector neurons. The interactions between these two critical neuronal properties along the tonotopic axis help create optimal ITD coding strategies dependent upon the frequency of the auditory inputs.

## AUTHOR CONTRIBUTIONS

Yong Lu conceived and supervised the study; William R. Hamlet and Yong Lu designed the experiments; William R. Hamlet, Yu-Wei Liu, Zheng-Quan Tang, and Yong Lu performed the research and analyzed data; William R. Hamlet and Yong Lu wrote the paper.

## Conflict of Interest Statement

The authors declare that the research was conducted in the absence of any commercial or financial relationships that could be construed as a potential conflict of interest.
